# Recent Advances in Omega-3: Health Benefits, Sources, Products and Bioavailability

**DOI:** 10.3390/nu6093727

**Published:** 2014-09-16

**Authors:** Peter D. Nichols, Alexandra McManus, Kevin Krail, Andrew J. Sinclair, Matt Miller

**Affiliations:** 1 Commonwealth Scientific Industrial Research Organization, Food and Nutrition Flagship, Oceans and Atmosphere Flagship, GPO Box 1538, Hobart, TAS 7000, Australia; 2 Centre of Excellence Science Seafood and Health, Curtin University, 7 Parker Place, Technology Park, WA 6102, Australia; E-Mail: A.McManus@curtin.edu.au; 3 The Omega-3 Centre, 32A Owen Street, North Bondi, NSW 2026. Australia; E-Mail: kkrail@omega-3centre.com; 4 Deakin University, School of Medicine, Waurn Ponds, VIC, 3217, Australia; E-Mail: andrew.sinclair@deakin.edu.au; 5 The New Zealand Institute for Plant & Food Research Limited, P.O. Box 5114, Nelson 7010, New Zealand; E-Mail: matt.miller@plantandfood.co.nz

**Keywords:** lipids, human health, aquaculture, long-chain omega-3, EPA, DHA

## Abstract

The joint symposium of The Omega-3 Centre and the Australasian Section American Oil Chemists Society; Recent Advances in Omega-3: Health Benefits, Sources, Products and Bioavailability, was held November 7, 2013 in Newcastle, NSW, Australia. Over 115 attendees received new information on a range of health benefits, aquaculture as a sustainable source of supply, and current and potential new and novel sources of these essential omega-3 long-chain (LC, ≥C_20_) polyunsaturated fatty acid nutrients (also termed LC omega-3). The theme of “Food *versus* Fuel” was an inspired way to present a vast array of emerging and ground breaking Omega-3 research that has application across many disciplines. Eleven papers submitted following from the Omega-3 Symposium are published in this Special Issue volume, with topics covered including: an update on the use of the Omega-3 Index (O3I), the effects of dosage and concurrent intake of vitamins/minerals on omega-3 incorporation into red blood cells, the possible use of the O3I as a measure of risk for adiposity, the need for and progress with new land plant sources of docosahexaenoic acid (DHA, 22:6ω3), the current status of farmed Australian and New Zealand fish, and also supplements, in terms of their LC omega-3 and persistent organic pollutants (POP) content, progress with cheap carbon sources in the culture of DHA-producing single cell organisms, a detailed examination of the lipids of the New Zealand Greenshell mussel, and a pilot investigation of the purification of New Zealand hoki liver oil by short path distillation. The selection of papers in this Special Issue collectively highlights a range of forward looking and also new and including positive scientific outcomes occurring in the omega-3 field.

## 1. Preface

The one day symposium; Recent Advances in Omega-3: Health Benefits, Sources, Products and Bioavailability, was co-convened by The Omega-3 Centre (O3C) and the Australasian Section of the American Oil Chemists Society (AAOCS) in Newcastle, Australia on November 7, 2013. The symposium was part of a three day Conference where over 150 scientists, researchers and industry representatives gathered for talks and discussions on a variety of lipid related topics. The Omega-3 Symposium was a full day devoted to presentations on recent advances in omega-3 research and was attended by over 115 scientists and industry representatives, with several additional presentations occurring the following day of the conference [1]. The Omega-3 Centre operates as a specialty healthcare association and center of excellence for omega-3 fatty acids for the Australia and New Zealand region. The primary focus of the O3C is to communicate the “good science” and health benefits of long-chain (≥C_20_) omega-3 (LC omega-3) oils, and to help translate the science and nutritional health benefits of omega-3 oils to key opinion leaders, including scientists, healthcare practitioners, the media and the public at large.

## 2. Summary of Papers in this Special Issue

Professor Clemens von Shacky from the Preventive Cardiology Unit at the Ludwig Maximilians-University of Munich opened the Omega-3 Symposium with a keynote address on the Omega-3 Index (O3I) as a biomarker of heart health [2]. He indicated that recent large trials with eicosapentaenoic acid (EPA) and docosahexaenoic acid (DHA) in the cardiovascular field did not demonstrate a beneficial effect in terms of reductions of clinical endpoints such as total mortality, sudden cardiac arrest or other major adverse cardiac events, and that pertinent guidelines do not currently uniformly recommend EPA + DHA for cardiac patients. His paper—Omega-3 Index and cardiovascular health—therefore emphasized the importance of limiting intervention trials to those people identified with low initial omega-3 status, use of the HS-Omega-3 Index^®^ methodology, and also ensuring that omega-3 supplements are consumed with a meal, as bioavailability data indicated that omega-3 uptake was dependent on concomitant fat intake.

Associate Professor Andrew Pipingas gave a presentation on neurocognitive benefits of omega-3 in healthy adults. In his co-authored paper—Randomized controlled trial examining the effects of fish oil and multivitamin supplementation on the incorporation of omega-3 and omega-6 fatty acids into red blood cells—healthy adult humans were randomized to receive 6 g of fish oil (1.8 g of EPA + DHA), 6 g of fish oil plus a multivitamin, 3 g of fish oil plus a multivitamin or a placebo daily for 16 weeks [3]. Treatment with 6 g of fish oil, with or without a daily multivitamin, led to higher red blood cell EPA composition at endpoint, with DHA composition unchanged. The O3I was only higher in the group receiving the combination of 6 g of fish oil and multivitamin. All treatments increased EPA incorporation in females while, in males, EPA was only significantly increased by the 6 g fish oil plus multivitamin combination. Considerable individual variability occurred in the red cell incorporation of EPA and DHA at endpoint. Gender contributed to a large proportion of this variability with females generally showing higher omega-3 composition at endpoint. It was concluded that the incorporation of LC omega-3 into red blood cells is influenced by dosage, the concurrent intake of vitamin/minerals and gender.

Professor Peter Howe presented data linking the O3I to prediction of risk for coronary heart disease [4]. As the index expresses circulating EPA + DHA as a percentage of total erythrocyte fatty acids, Professor Howe posited that this could be a novel way to measure not only the risk of heart disease, but other common health conditions such as cognitive decline and mental health disorders. The main focus of this paper was the possible use of the O3I as a measure of risk for adiposity. A review of research into adiposity, body composition and erythrocyte EPA and DHA in both men and women found that the low levels of DHA in women were predictive of adiposity risk. There was no associated risk with EPA levels in men. These findings indicate that the O3I could be a quick and cost effective clinical marker for assisting the risk of adiposity in women. Furthermore, this study supports the trial of adequate intakes of DHA as a complimentary therapy for the management and treatment of overweight individuals and obesity.

The review paper by Dr. Welma Stonehouse from CSIRO covered the topic that LC omega-3 derived from marine sources may play an important role in cognitive performance throughout all life stages [5]. DHA, the dominant omega-3 in the brain, is a major component of neuronal cell membranes and affects various neurological pathways and processess. Despite its critical role in brain function, human’s capacity to synthesize DHA *de novo* is limited and its consumption through the diet is therefore essential. However, many individuls do not or rarely consume seafood. Dr. Stonehouse critically evaluated evidence from randomised controlled trials (RCT) in healthy school-aged children, younger and older adults to determine whether consumption of LC omega-3 PUFA improves cognitive performance and to make recommendations for future research. Study design limitations in many RCTs hamper firm conclusions. The measurement of a uniform biomarker, e.g., %DHA in erythrocytes, is essential to establish baseline DHA-status, to determine targets for cognitive performance and to facilitate dosage recommendations. It was recommended that future studies be at least 16 weeks in duration, account for potential interaction effects of gender, age and apolipoprotein E genotype and include measures of speed of cognitive performance.

Several presentations at the symposium covered new sources of LC omega-3 oil, including discussing exciting developments with new land plant sources of long-chain omega-3 oils. A review paper by Dr. Soressa Kitessa and colleagues from CSIRO examined use of oils containing the shorter-chain omega-3, stearidonic acid (SDA, 18:4ω3), in a range of livestock and fish feeding trials with lamb, chicken, Atlantic salmon and barramundi [6]. Interest in the use of SDA has been enhanced by the development of SDA-containing genetically modified soyabean oil which is planned to soon enter the US market. However, neither oils from traditional oilseeds such as linseed, nor the new SDA soyabean oil have demonstrated efficient conversion to DHA in the animals’ trialed or in humans. It is this knowledge that has driven the quest by a number of research groups to produce oil seeds containing LC omega-3, in particular DHA. Previous attempts to produce DHA in oilseeds only achieved low levels of DHA and also were high in omega-6 PUFA and contained a high omega-6/omega-3 ratio. Dr. Surinder Singh in his conference presentation [1] and Dr. Kitessa and colleagues [6] described a recent breakthrough that has demonstrated the ability to produce land plant-based oil particularly enriched in DHA, with low omega-6 PUFA levels, and an omega-3 to omega-6 ratio close to that occurring in marine oils/seafood [7]. Therefore, the future availability of land plant oils containing both EPA and DHA can supplement the demand for marine sources of LC omega-3 oils in a range of areas. This in turn will enhance the sustainability of global fisheries, enable the consumer to meet the recommended dietary targets for these oils, assist in aquaculture nutrition and the development of an innovative food and feed industry, and ultimately deliver improved health of consumers.

The paper by Dr. Peter Mansour and colleagues from CSIRO—Characterization of oilseed lipids from DHA-producing *Camelina sativa*: A new transformed land plant containing long-chain omega-3 oils—provided detailed lipid class and fatty acid profiles for a new land plant derived oil [8]. Triacylglycerols (TAG) were the major lipid class in hexane extracts. Chloroform-methanol (CM) extraction recovered further lipid comprising glycolipids and phospholipids and residual TAG. The main phospholipid species were phosphatidyl choline and phosphatidyl ethanolamine. The % DHA was: 7% (of total fatty acids) in the TAG-rich hexane extract and 4% in the polar lipid-rich CM extract. The relative level of ALA in DHA-containing *Camelina* seed was higher than the control. Sterols and fatty alcohols were characterized, with iso-branched odd-chain fatty alcohols, also present.

Several members from the Centre for Chemistry and Biotechnology lead by Professor Colin Barrow at Deakin University covered aspects of single cell oil production. A paper by Thyagarajan and colleagues examined—Evaluation of bread crumbs as a potential carbon source for the growth of Thraustochytrid species for oil and omega-3 production [9]. The utilization of food waste by microorganisms to produce omega-3 fatty acids or biofuel is a low cost method with environmental benefits. It was shown that the marine microorganisms *Thraustochytrium* sp. AH-2 and *Schizochytrium* sp. SR21 were able to use breadcrumbs as an alternate carbon source for the production of lipids under static fermentation conditions. Liquid fermentation of *Thraustochytrium* sp. AH-2 with glucose produced 4.3 g/L of biomass and 44 mg/g of saturated fatty acids after seven days. Static fermentation of both species with breadcrumbs resulted in 2.5 g/L and 4.7 g/L of biomass, and 42 mg/g and 34 mg/g of saturated fatty acids, respectively. Scanning electron microscopic studies confirmed the growth of both strains on breadcrumbs. Attenuated total reflection Fourier transform infrared spectroscopy findings for *Schizochytrium* sp. SR21 were consistent with the utilization of breadcrumbs for the production of unsaturated lipids, albeit at relatively low levels. The total lipid yield for static fermentation with bread crumbs was marginally lower than for fermentation with glucose media, while the yield of unsaturated fatty acids was considerably lower, indicating that static fermentation may be more appropriate for the production of biodiesel than for the production of omega-3 rich oils in these strains.

Dr. Matthew Miller and colleagues from Plant and Food, New Zealand described the distribution of lipids in Greenshell™ Mussel (GSM) (*Perna canaliculus*) [10]. He indicated that GSM are a sustainable source of omega-3 LC-PUFA, as they require no dietary inputs, gaining all of their nutrient requirements by filter-feeding microorganisms from sea water. GSM oil is valued considerably higher than fish oils, and has been reported to have important health benefits, for example, anti-inflammatory activity. It contains several minor lipid components—such as non-methylene interrupted FA, plasmalogens and phytosterols—that are not present in most fish oil products. The lipid content of the female GSM was shown to be significantly greater than that of the male, and the major lipid class in both genders was phospholipid. Female GSM contained more LC omega-3, and stored a greater proportion of total lipid in the gonad and mantle. The higher lipid content in the female was likely related to gamete production. The mantle and digestive gland were other important sites for lipid storage and/or function/production.

Seafood continues to be one of the major sources of LC omega-3 oils. Following an update by Professor Giovanni Turchini on the use of omega-3 in aquaculture [1], Dr. Peter Nichols and co-authors provided a presentation: Readily available sources of long-chain omega-3 oils: Is farmed Australian seafood a better source of the good oil than wild-caught seafood? [11]. The two major farmed Australian finfish species, Atlantic salmon and barramundi, have higher oil and LC omega-3 content than the same or other species from the wild, and remain an excellent means to achieve substantial intake of LC omega-3 oils. Notwithstanding, LC omega-3 oil content has decreased in these two farmed species, due largely to the replacing of dietary fish oil with poultry oil in the feed. For Atlantic salmon, LC omega-3 content decreased ~30%–50% between 2002 and 2013, and the omega-3/omega-6 ratio also decreased (>5:1 to <1:1). The development and future application of oilseeds containing LC omega-3 oils and their incorporation in aquafeeds would allow these health-benefitting oils to be maximized in farmed Australian seafood. As Australian consumers increasingly seek their LC omega-3 from supplements, a range of supplement products were also compared; all products met their label specifications, with considerable variation occurring in relative levels of EPA and DHA and the cost to consumers for consumption of 500 mg of EPA + DHA per day.

Adam Ismail from the Global Organization for EPA and DHA Omega-3 (GOED) in a plenary lecture at the Omega-3 Session presented a challenge to the fish oil industry around demand and supply [1]. He highlighted the Omega-3 ingredient marketplace as a growing US $25 billion industry, with an increase in krill and new pharmaceutical omega-3 products predicted to gain significant market penetration which will put even further demand pressure on resources.

Building on the theme of Adam Ismail, increasing the quality of products derived from smaller fishers will also help with the supply issue; work presented by the collaboration of Dr. Alex Oliveria (Kodiak, AK, USA) and Dr. Matt Miller (Nelson, New Zealand) used short path distillation as a method to improve the quality of the Alaskan pollock (*Gadus chalcogrammus*) and New Zealand’s hoki (*Macruronus novaezelandiae*) oils [12]. This paper—Purification of Alaskan walleye pollock (*Gadus chalcogrammus*) and New Zealand hoki (*Macruronus novaezelandiae*) liver oil using short path distillation—demonstrated that this technology could significantly enhance oil quality parameters (free fatty acids, peroxide and *para*-anisidine values), and that purified oils obtained met the GOED standard for edible fish oils.

The paper by Susan Bengtson Nash, University of Griffiths, and colleagues addressed: The nutritional-toxicological conflict associated with fish oil *versus* Antarctic krill oil dietary supplements [13]. Fish oil supplements and complementary medicines play a role of increasing importance in meeting daily requirements of essential nutrients such as LC omega-3 and Vitamin D. A new product category, derived from Antarctic krill, has gained an increasing share of the omega-3 nutriceutical market. Antarctic krill oil is marketed as demonstrating a greater ease of absorption due to higher phospholipid content, as being sourced through sustainable fisheries and being free of toxins and pollutants. However, limited data is available on the latter. Persistent Organic Pollutants (POP) encompasses a range of toxic, man-made contaminants that accumulate in marine ecosystems and in the lipid reserves of organisms. The study provides the first quantitative comparison of the nutritional (EPA and DHA) *versus* the toxicological profiles of Antarctic krill oil products relative to other fish oil categories on the Australian market. Krill oil products adhered closely to EPA and DHA manufacturer specifications and contained intermediate levels of POP when compared to other products. Monitoring of the pollutant content of fish and krill oil products will become increasingly important with expanding regulatory specifications for chemical thresholds.

The selection of papers in this Special Issue highlights a range of new and including positive scientific outcomes occurring in the omega-3 field across the areas of health benefits, sources, products and bioavailability. Future research in the Australasia region will continue in these areas and will assist in further increasing our understanding of the key health-benefitting long-chain omega-3 oils.
